# FAST Heroes: Results of Cross-Country Implementation of a Global School-Based Stroke Education Campaign

**DOI:** 10.3389/fpubh.2022.849023

**Published:** 2022-04-18

**Authors:** Kalliopi Tsakpounidou, Jan van der Merwe, Marianne Elisabeth Klinke, Chris Webb, Sheila Cristina Ouriques Martins, Hariklia Proios

**Affiliations:** ^1^Department of Educational and Social Policy, University of Macedonia, Thessaloniki, Greece; ^2^Boehringer Ingelheim International Gesellschaft mit Beschränkter Haftung (GmbH), Healthcare Affairs and Patient Engagement, Ingelheim am Rhein, Germany; ^3^Faculty of Nursing, School of Health Sciences, University of Iceland, Reykjavik, Iceland; ^4^Twelve, Worthing, United Kingdom; ^5^Hospital de Clínicas de Porto Alegre, Universidade Federal do Rio Grande do Sul, Hospital Moinhos de Vento, Neurology, Porto Alegre, Brazil

**Keywords:** children, education, knowledge, stroke, symptoms, pre-hospital care

## Abstract

**Background:**

Educating the at-risk population about stroke symptoms and requirement of calling an ambulance when stroke strikes is challenging. This exploratory cross-country study provides insights to the FAST Heroes educational campaign and outcomes hitherto achieved.

**Aims:**

The primary aim of the study was to measure the transfer of stroke-related knowledge to parents after a global school-based FAST Heroes educational campaign for 5- to 9-year-old children in 14 different countries. The secondary aim was to evaluate parents and teachers' acceptability toward the program.

**Methods:**

The duration of the program was 5 h; 1 h per week, joining face-to-face educational sessions with workbooks, cartoons, web-based learning, and other fun activities. Outcomes were measured before implementation (t1), after implementation (t2), and at 6-month follow-up (t3). Program acceptability and stroke knowledge were evaluated by feedback surveys for teachers and parents.

**Results:**

Worldwide, 4,202 parents completed the program with their children and answered surveys at t1 and t2. They increased their knowledge of three stroke symptoms from 48 to 83% (*p* < 0.001). All three surveys were completed by 86 parents, who improved their knowledge of stroke symptoms, 55% (t1), 79% (t2), and 94% (t3) (*p* < 0.001). Overall, the educational messages were successfully passed onward.

**Conclusions:**

Findings confirm the primary aim of the study that is knowledge about stroke transfer well from children to their families through the FAST Heroes program. Second, parents and teachers globally consider the program feasible and worthwhile. The results will inform further rollout of the campaign.

## Introduction

Stroke is the second leading cause of death worldwide and the leading cause of acquired disability in Europe (1). The number of persons aged 60 or above is expected to more than double from 2017 to 2050 (2). Consequently, this will lead to an approximate 34% increase in stroke incidence from 2015 to 2035 (3). Early treatment for ischemic stroke decreases the likelihood of disability within three months by at least 30% (4, 5). Successful medical treatment is time-dependent and should preferably be administered within a 4.5-h window (6–8). Research shows that as few as 15% of stroke patients arrive at the hospital within this timeframe (9). Late hospital arrival from stroke onset has been associated with certain social determinants like higher age and race. Some ethnic groups have lower stroke awareness than others (10). On the other hand, existing comorbidities, such as stroke history and related risk factors (e.g., hypertension, atrial fibrillation) play an important role in earlier response (7, 10–13). Consequently, it is not just the knowledge of stroke symptoms that prompt timely hospital arrival but also the response to symptoms (12, 14). It is thus crucial that people correctly identify symptoms pointing toward a stroke that they or their loved ones encounter (15). Furthermore, the perceived danger of symptoms is a motivating factor for persons with a suspected stroke to seek immediate help. Unfortunately, only 11% of stroke patients call an ambulance instantly after symptom onset, 67% opt to call a relative or family member as their first response, while 70% of the people from whom advice is solicited do not recommend calling an ambulance “now” (11). These striking results emphasize an urgent need for launching effective national and international educational campaigns to improve symptom-to-treatment times.

To understand how to educate the public about stroke, one first needs to understand whom to educate. The median adjusted age at which the first stroke occurs is 70.3 years in Europe (with an interquartile range of 14.1 years) (16, 17). Concurrently, the Eurostat Ageing Europe report shows that as many as 54.1% of people aged 50–64, the “Baby Boomers,” spend several days a week caring for their grandchildren (18, 19). On that account, the Health Promoting Schools approach (20) suggested that a significant advantage of using schools and children for health promotion has the potential to reach a large part of the community, such as grandparents, through a single channel because more than 90% of children worldwide are in school (21).

Over the last two decades, several health-related campaigns targeting children have shown impressive results (22–27). The Head Start program in the US concluded that children embracing healthy behaviors transfer this knowledge to their extended family. They advocate for a healthy diet, encourage parents to stop smoking, and favor face-to-face activities over more screen time. The family unit with young children can become an important pylon for adopting more healthy living habits. They showed that ambulance visits declined by 58%, and school attendance was reduced by 29%. Days absent from work for parents were decreased by 42% (28). Another study showed that only 3% of parents in the intervention group identified all four letters of the stroke FAST (Facial droop, Arm weakness, Speech disturbance, Time to call 911) acronym before their training. Knowledge of FAST increased to 20% at immediate post-training and 17% at 3-month delayed post-training (29).

The FAST acronym was developed in the UK in 1998 and has been culturally adapted worldwide. Studies using FAST have demonstrated high accuracy in diagnosing strokes by paramedics and emergency medical technicians with positive predictive values between 61 and 77% (30). The “Grand Mission” FAST Heroes project is a global school-based stroke educational campaign that educates children and their extended family (i.e., parents/guardians, grandparents) about stroke symptomatology and the necessary actions in response to stroke. FAST Heroes program differs significantly from other programs because it targets kindergarten children from the age of 4 years old, and it aims to educate the extended family, outside the nuclear family, about stroke symptom recognition and appropriate course of action. In this way, stroke knowledge can be delivered to the broader community (31, 32). The element that is added in this study is the transference of stroke knowledge from children to their parents within an international setting. Despite regional variations, we look at the compiled effects of the Grand mission FAST Heroes' program across multiple countries.

The primary aim of this descriptive cross-country study is to disseminate the first results of the global “Grand Mission” FAST Heroes program and its effectiveness on knowledge transfer to parents of participating children worldwide. Researchers have recently referred to “stroke knowledge” as knowledge of stroke warning signs, and appropriate behavioral response to stroke symptoms (33, 34). In addition to our primary aim, we also examine the program's impact 6 months after implementation to evaluate trends regarding long-term knowledge attrition. The secondary aim of the study is to report parents' and teachers' perceptions about the global campaign.

## Materials and Methods

The FAST Heroes campaign includes a comprehensive set of educational resources that was designed and developed in 2018 by a multidisciplinary team consisting of kindergarten teachers, health care professionals, school psychologists etc. A series of pilot studies were used to assess children's baseline stroke knowledge and fine-tune the educational approach for 5- to 9-year-old children so they carry their stroke-related knowledge onwards to their parents For further details please see (31, 32, 35). Among other teaching aids, a website^1^ was created in order to involve parents in the educational process. We used the relevant local emergency number as a mental peg and linked the stroke symptoms to this number (36, 37). We adapted learning theories, e.g., spacing, repetition, and pegging techniques, to refresh knowledge, overcome the forgetting curve, and assisting children in systematically internalizing the knowledge (38). We also created a direct communication channel to the Grandparents by using principle learning strategies such as incidental learning through their grandchildren (39, 40). These studies yielded promising results regarding increased knowledge and stroke preparedness for children and their families.

The successful implementation of the program on a national level in Greece justified its expansion globally. To date, the campaign has been adapted to 11 local emergency numbers, i.e., 103, 106, 107, 112, 123, 131, 192, 911, 995, 998, 999. It has also been translated into the local language for a still growing list of more than 37 communities, including Argentina, Belarus, Brazil, Bulgaria, Canada, Catalonia, Chile, Colombia, Croatia, Czech Republic, Ecuador, Egypt, Georgia, Germany, Greece, Hungary, Iceland, Italy, Iran, Kazakhstan, Lithuania, Malaysia, Moldova, Peru, Poland, Portugal, Romania, Russia, Singapore, Slovakia, South Africa, Spain, Ukraine, United Arab Emirates, United Kingdom, and Uzbekistan. From March to June 2021, a global initiative called “Grand Mission” was launched, through which we aim to enroll one million children and their families worldwide in the next 5 years (41). Public Relations (PR) agencies were recruited in every participating country to assist with the Grand Mission project implementation as well as recruit local, national and international celebrities and social media influencers for promotional activities.

The project received endorsement of the World Stroke Organization (July 20, 2019), Stroke Alliance for Europe (February 11, 2019), and the Schools for Health in Europe Network Foundation (June 23, 2020). This study received clearance from the Committee for Research Ethics of the University of Macedonia (Thessaloniki, Greece) (14/15.06.2020), where the program's educational content was developed. The ethical permission is in accordance with the 1964 Declaration of Helsinki.

### Study Design

**Figure 1:**
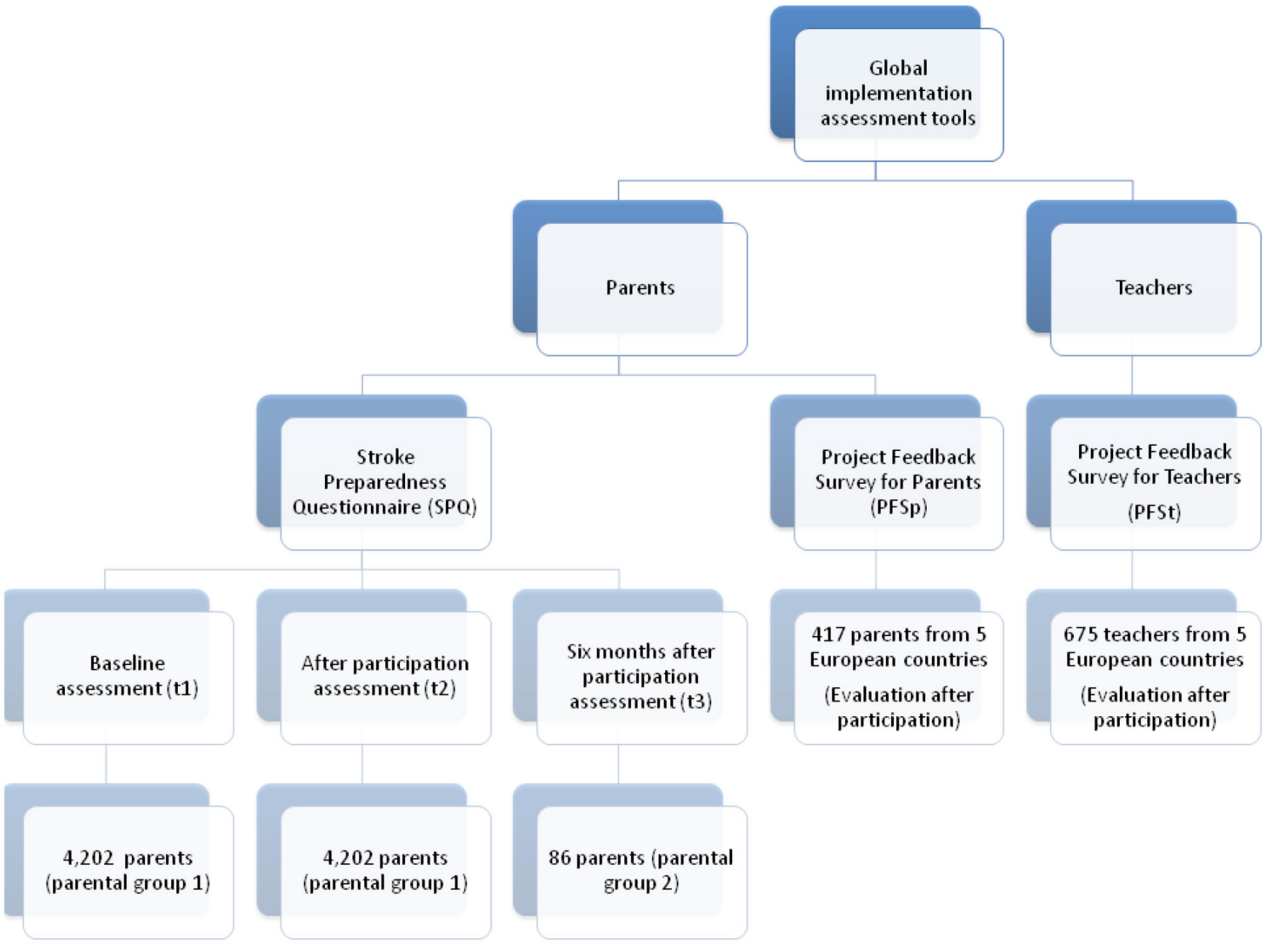
Flowchart describing the assessment tools used for parents and teachers' participation.

Three assessment tools were used in the present study in total (Figure 1). Parents' stroke knowledge was evaluated by employing an online assessment using the Stroke Preparedness Questionnaire (SPQ) in three phases of the program. Test 1 (t1) evaluated their baseline knowledge ahead of participating in the program. Test 2 (t2) measured the knowledge gains immediately after participation in the program, and Test 3 (t3) measured acquired knowledge 6 months after participation. The SPQ is available online on the FAST Heroes website to all registered families (Supplementary Material Data I). In order to complete their registration and to collect points so that they get certificate participation for their children, parents had to complete the SPQ in all three phases. The SPQ is self-administered, and all questions are a modified from previous questionnaires (42). All materials were translated and adapted to each country's culture and context. After a standard forward-backward translation procedure from English to each country's language by a certified translator, the translation was checked by a research team member to ascertain that the stroke message was rendered correctly.

To measure the campaign's acceptance level among participating teachers and parents, we designed surveys for each group, namely Project Feedback Survey for Teachers (PFSt) and Project Feedback Survey for Parents (PFSp) and participants were directed toward it *via* email to complete it voluntarily. The PFSt and PFSp survey consisted of 27 and 28 questions, respectively (Supplementary Material Data II) and hosted *via* an online portal managed by 3 Gem Research^2^ Participants were directed toward these two surveys *via* email.

### Participants

All parents who had registered on the website with their children participated in the SPQ assessment. Due to different starting dates of the program in each country, parents were divided into 2 groups: Parental group 1 consists of parents who completed the two assessments of the SPQ (t1 and t2), group 2 consists of parents who completed the SPQ in all three assessments (t1, t2, and t3). Teachers and parents from Greece, Poland, Romania, Slovakia, and Ukraine, which were the countries with the most schools participating in the program, were targeted to measure their level of satisfaction and acceptance of the campaign.

### Statistical Analysis

IBM SPSS Statistics 25 software was used. The McNemar test for related samples examined the relation of parents' answers in t2 and t3 as regards stroke symptoms (question 2, i.e., “Which of the following do you think could be a symptom of a stroke?”) and the selected course of action in the event of witnessing a stroke (question 1, i.e., “Please read through the symptoms listed on the left and tell us what you would do in each case if someone with you is showing that particular symptom”). A Chi-Square test examined whether the educational program impacted parents' knowledge in relation to choosing the corresponding emergency number in each country in t2 and t3 (question 3, i.e., “Do you know the number to use to call an ambulance?”). All tests were conducted at the α = 5% significance level.

## Results

### Parental Group 1

Parental group 1 comprises parents who completed the campaign and the assessments at t1 and t2 (*n* = 4,202). At t1, 32% of parents chose “Call an ambulance” for all three symptoms vs. 70% at t2. Almost half of the parents (48%) were able to identify all three stroke symptoms at t1 vs. 83% at t2 (Table 1). Speech disturbance was the most recognizable stroke symptom in t1 and t2 (57 and 94%, respectively). At t1, 51% of parents correctly answered 112, 103, or 911, according to their country's designated emergency number, vs. 85% at t2 (Table 2). Regional variations in both the before and after results for stroke and emergency number knowledge were observed. The relative impact of the campaign remains high with the majority of countries showing growth in knowledge in excess of 100% (Table 3).

**Table 1:** Percentage of participants in parental group 1 who selected 1, 2, or 3 correct answers in the SPQ in t1 and t2.

***N* = 4,202**	**t1 (%)**	**t2 (%)**	**Absolute change (%)**	**Relative change in t1 and t2 (%)**	***p*-value**
**Question 1^a^**					
At least 1 correct	51	90^*^	39	77	>0.001
At least 2 correct	43	81^*^	38	88	>0.001
All 3 correct	32	70^*^	38	119	>0.001
**Question 2^b^**					
At least 1 correct	59	98^*^	39	66	>0.001
At least 2 correct	56	84^*^	28	50	>0.001
All 3 correct	48	83^*^	35	73	>0.001
**Question 3^c^**					
Correct number	51	85^*^	34	67	>0.001

* *Numbers in bold show the McNemar Test results (Question 1 and Question 2) for statistically significant difference and the Chi-square Test results (Question 3) between before and after parental group 1 participation who responded to the SPQ in t1 and t2*.

a *Question 1 refers to stroke and non-stroke symptoms and the appropriate steps that should follow each of them*.

b *Question 2 refers to stroke and non-stroke symptoms among which participants were asked to choose the stroke symptoms*.

c *Question 3 refers to the culturally-appropriate emergency number to be called in case of a stroke*.

**Table 2:** Percentage of correct answers from participants in parental group 1 who responded to the SPQ in t1 and t2.

***N* = 4,202**	**t1 (%)**	**t2 (%)**	**Absolute change (%)**	**Relative change in t1 and t2 (%)**	***p*-value**
**Question 1^a^**					
Scenario with Face symptom	40	78^*^	38	95	>0.001
Scenario with Arm symptom	43	81^*^	38	88	>0.001
Scenario with Speech symptom	44	83^*^	39	89	>0.001
**Question 2^b^**					
Face symptom	55	93^*^	38	69	>0.001
Arm symptom	51	87^*^	36	71	>0.001
Speech symptom	57	94^*^	37	65	>0.001

* *Numbers in bold show the McNemar Test results for statistically significant difference between before and after parental group 1 participation who responded to the SPQ in t1 and t2*.

a *Question 1 refers to stroke and non-stroke symptoms and the appropriate steps that should follow each of them*.

b *Question 2 refers to stroke and non-stroke symptoms among which participants were asked to choose the stroke symptoms*.

**Table 3:** Country breakdown of percentage of respondents who indicated the correct course of action for all three stroke symptoms in question 1 and 3- in parental group 1 (who responded to the SPQ in t1 and t2).

**Country**	**Before—Q1^a^ (%)**	**After—Q1 (%)**	**Absolute change (%)**	**Relative change (%)**	**Before—Q3^b^ (%)**	**After—Q3 (%)**	**Absolute change (%)**	**Relative change (%)**
Brazil (*n* = 96)	39	77	38	97	58	92	34	59
Bulgaria (*n* = 42)	24	57	33	138	45	86	41	91
Greece (*n* = 367)	21	54	33	157	31	68	37	119
Italy (*n* = 64)	34	56	22	65	81	92	11	14
Lithuania (*n* = 180)	42	74	32	76	57	90	33	58
Poland (*n* = 327)	22	66	44	200	28	80	52	186
Romania (*n* = 426)	33	69	36	109	54	90	36	67
Slovakia (*n* = 624)	42	77	35	83	54	88	34	63
South Africa (*n* = 46)	20	63	43	215	43	87	44	102
Spain (*n* = 142)	30	77	47	157	80	94	14	18
Ukraine (*n* = 1,733)	34	71	37	109	56	88	32	57

a *Question 1 refers to stroke and non-stroke symptoms and the appropriate steps that should follow each of them*.

b *Question 3 refers to the culturally-appropriate emergency number to be called in case of a stroke*.

The McNemar test revealed that the change in question 1 and 2 between t1 and t2 was significant (p <0.001). A Chi-Square test showed that the educational program had a significant effect regarding the emergency number respondents would select to call an ambulance (question 3) (p <0.001).

### Parental Group 2

**Figure 2:**
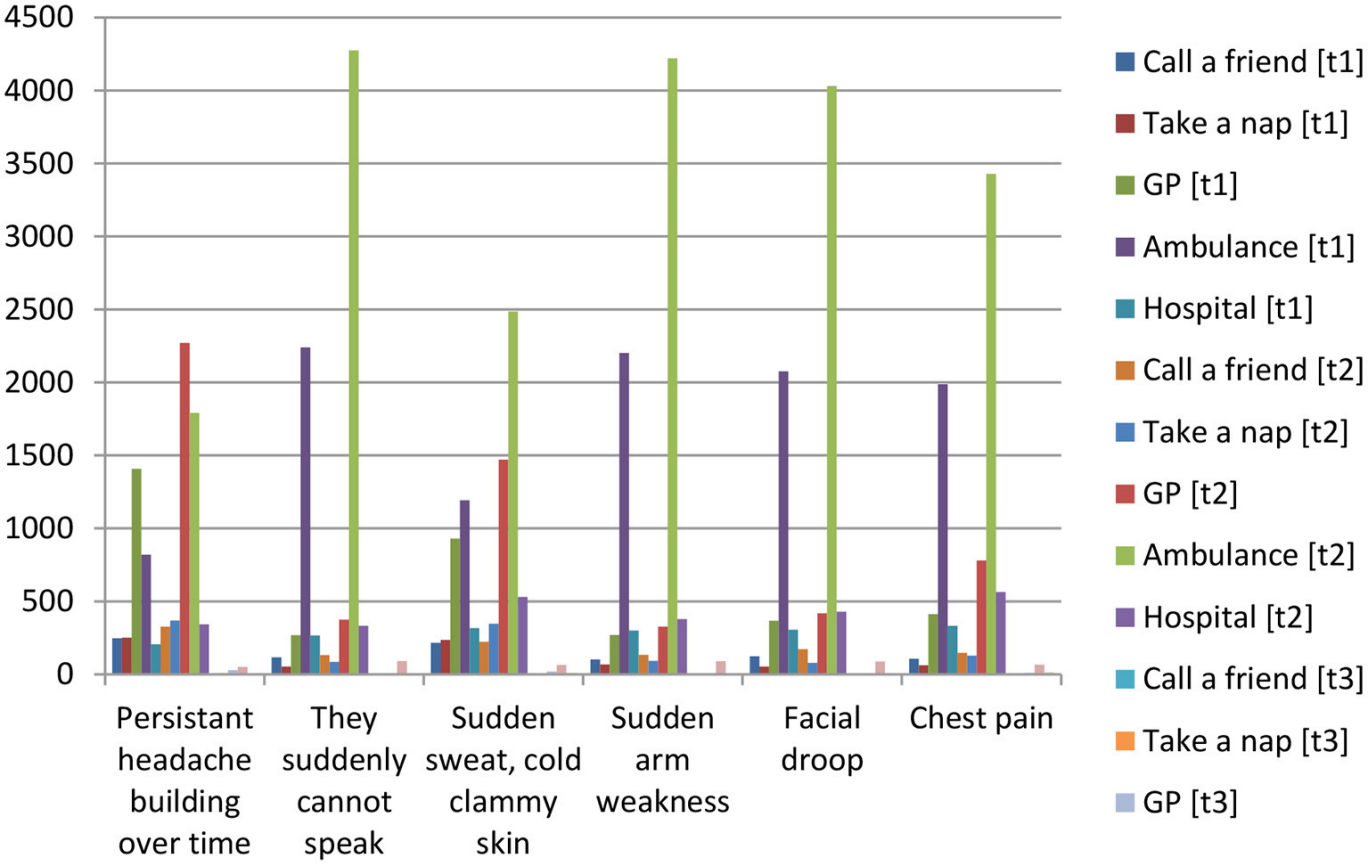
Graph of parents' answers in regard to the action they would take in case of stroke and non-stroke symptoms across the three assessments (t1, t2, t3).

Parental group 2 comprises parents who completed the program and assessments at t1, t2, and t3 (*n* = 86 parents). At t1, 30% of parents chose the correct response for all three symptoms, 65% (t2) and to 83% (t3) (Table 4). More than half of the parents (55%) were able to identify all three stroke symptoms before implementation. This increased to 79% (t2) and to 94% (t3). Fifty-five percent (55%) of participants correctly answered 112, 103, or 911 (t1), vs. 85% (t2) (Table 5). All parents (100%) recalled the correct emergency number at 6 months. The McNemar test showed a significant increase in recognition of all three symptoms for question 2 between t1 and t3 (*p* < 0.001). Figure 2 illustrates parents' answers in regard to the action they would take in case of stroke and non-stroke symptoms across the three assessments.

**Table 4:** Percentage of participants in parental group 2 who selected 1, 2, or 3 correct answers in the SPQ in t1, t2, and t3.

***N* = 86**	**t1 (%)**	**t2 (%)**	**t3 (%)**	**Absolute change t2 (%)**	**Absolute change t3 (%)**	**Relative change t2 (%)**	**Relative change t3 (%)**	***p*-value**
**Question 1^a^**								
At least 1 correct	58	83	93	25	35	43	60	0.067
At least 2 correct	43	77	90^*^	34	47	79	109	0.023
All 3 correct	30	65	83^*^	35	53	117	177	0.019
**Question 2^b^**								
At least 1 correct	72	97	100^*^	25	28	35	39	0.045
At least 2 correct	65	92	99^*^	27	34	42	52	0.032
All 3 correct	55	79	94^*^	24	39	44	71	0.026

* *Numbers in bold show the McNemar Test results for statistically significant difference between before and 6 months after parental group 2 participation*.

a *Question 1 refers to stroke and non-stroke symptoms and the appropriate steps that should follow each of them*.

b *Question 2 refers to stroke and non-stroke symptoms among which participants were asked to choose the stroke symptoms*.

**Table 5:** Percentage of correct answers from participants in parental group 2 who responded to the SPQ in t1, t2, and t3.

***N* = 86**	**t1 (%)**	**t2 (%)**	**t3 (%)**	**Absolute change t2 (%)**	**Absolute change t3 (%)**	**Relative change t2 (%)**	**Relative change t3 (%)**	***p*-value**
**Question 1^a^**								
Scenario with Face symptom	40	71	87	31	47	78	118	0.082
Scenario with Arm symptom	43	77	88	34	45	79	105	0.051
Scenario with Speech symptom	49	77	90^*^	28	41	57	84	0.018
**Question 2^b^**								
Face symptom	66	92	99^*^	26	33	39	50	0.005
Arm symptom	60	85	95^*^	25	35	42	58	0.014
Speech symptom	65	91	99^*^	26	34	40	52	0.009
**Question 3^c^**								
Correct	55	85	100^*^	30	45	55	82	0.019

* *Numbers in bold show the McNemar Test results for statistically significant difference between before and 6 months after parental group 2 participation*.

a *Question 1 refers to stroke and non-stroke symptoms and the appropriate steps that should follow each of them*.

b *Question 2 refers to stroke and non-stroke symptoms among which participants were asked to choose the stroke symptoms*.

c *Question 3 refers to the culturally-appropriate emergency number to be called in case of a stroke*.

### Project Feedback Surveys as Completed by Teachers and Parents

PFS was completed by 675 teachers and 417 parents from Poland, Slovakia, Romania, Greece, and Ukraine. In regard to the teachers, 37.5% of them implemented the educational program with 11–20 children, while 33.2% of them implemented the program in classes of 21–30 children. Schools were mainly located in towns or cities with fewer than 20,000 inhabitants (41.2%). The program was presented face-to-face by 59% of the teachers, whereas 224 teachers (33.2%) used a hybrid model and 53 teachers (7.8%) implemented all modules virtually. “*Supporting an important cause that can make a positive difference*” was selected by 85% of teachers for participating in the program, 64% of teachers selected “*It can help spread an important message”*, and 40% selected “*It looked interesting and rewarding for children”*. Some teachers (38.4%) knew a close relative or family friend who had suffered a stroke. Also, teachers agreed or strongly agreed that the campaign could help save lives (97.3%), that knowledge transfer had taken place (93.4%), that the children enjoyed taking part in the campaign (97.4%), and that the materials were of high quality (96%). “*The characters/FAST Heroes”* ranked the highest, followed by “*the animated films”, “the way they make learning fun”*, “*the online e-books”* and “*the educational workbooks”*. Almost the two thirds (62.5%) of teachers received unsolicited positive feedback from parents. The vast majority (98.1%) agreed or strongly agreed that they would recommend the campaign to others and 98.6% reported that the campaign had increased their confidence about what to do if somebody had a stroke. Table 6 summarizes teachers' demographic information.

**Table 6:** Demographic information of teachers who completed the Project Feedback Survey (PFSt).

	**Poland (*n* = 77)**	**Slovakia (*n* = 108)**	**Romania (*n* = 187)**	**Greece (*n* = 91)**	**Ukraine (*n* = 212)**	**Total (*n* = 675)**
**Gender**
Male	2	1	7	3	13	13
Female	77	106	186	84	209	662
**Age**
30 or under	8	10	11	16	37	82
31–40	24	25	50	22	70	191
41–50	28	41	90	21	73	253
51–60	17	27	36	31	32	143
Above 60	-	5	-	1	-	6
**Educational level**
Primary school	-	-	17	1	-	18
High school	-	9	4	1	11	25
University graduate	14	99	83	53	126	375
MSc or PhD	63	-	83	36	75	257

Sixty one percent of the children in the survey were 5–9 years old and 43% were >9 years old. Thirty one percent of parents in our survey had parents or parents-in-law living with them and 38.1% had a close relative or family friend who had suffered a stroke. Parents that agreed or strongly agreed that their children enjoyed taking part in the campaign (94.5%), that their child understood the message (93%), that their children had passed on the messages to their family members (86.8%). Parents agreed or strongly agreed that the campaign increased their own confidence about what to do when somebody had a stroke (91.4%), that the campaign could help save lives (93.7%), and that they would recommend the campaign to others (91.8%).

## Discussion

Process and outcome evaluations of the global FAST Heroes campaign were explored in this study in conjunction with the effectiveness of strategies used for its development and implementation. We examined the extent to which the campaign succeeded to meet its primary objective, that was evaluating the program's impact on knowledge delivery from children to their parents, as well as its secondary objectives, which included reporting parents' and teachers' perceptions about the campaign.

Results from parental group 1 who completed the SPQ in t1 and t2 of the study showed that they chose “Call an ambulance” for all three stroke symptoms in t2 which was a significant increase. Marto et al. (43) had similar results with parents more correctly regarding calling an ambulance if someone presented with stroke symptoms (43). Our findings confirm what is reported in the literature insofar as they show that school-based educational programs can be an effective, intergenerational model to enhance parental stroke literacy (34, 43–48). Parallel to previous studies the school-based educational programs appear to be both feasible and effective and the intergenerational model excellent for enhancing parental stroke literacy (49). A statistically significant improvement was found in parents' correct answers for the emergency number. Interestingly, a considerable cross-country variation existed among parents who indicated that they would choose the correct course of action in relation to all three stroke symptoms after the campaign ended from 54 to 77%. Regional differences within countries in terms of socioeconomic as well as educational levels could partly explain this finding. However, this issue merits attention in future studies.

Stroke literacy of parents has been examined up to 3 months post-intervention (49). Knowledge retention through the FAST Heroes educational program extended even further, or until 6 months after program completion. Importantly, parents found it harder to choose the correct action (call an ambulance) when presented with stroke symptoms mixed with non-stroke symptoms vs. being asked to select stroke symptoms from a preselected symptom list. This finding explains the unbridged gap between recognizing stroke symptoms or considering stroke a medical emergency and the lack of an appropriate response, i.e., seeking help. According to Mackintosh et al. (50), while the general public support that they would call an ambulance in case of a stroke, in reality both patients and bystanders often contact a general practitioner at first, which significantly delays medical care administration.

Teachers from 5 European countries (Poland, Slovakia, Romania, Greece, and Ukraine) participated in the current study. Nearly all teachers (98.1%) would recommend the campaign to others. Almost half of the teachers delivered parts of the program remotely. Due to local lockdown restrictions during the COVID-19 pandemic, almost half of the teachers delivered parts of the program remotely using online learning tools. This suggests that the program can be implemented both in class and digitally and may thus be viable to implement even in remote areas. Most teachers (90%) also recognized that children had fun while participating and that the educational material contained entertaining ways of transferring knowledge between children and their families.

Altogether, results concur with the primary goal of the FAST Heroes campaign, which is to educate young children and their extended families to recognize the main stroke symptoms and act urgently in the event of a stroke.

### Limitations and Future Studies

The descriptive exploratory nature of the study made it impossible to provide firm conclusions regarding causality. A recent systematic review and meta-analysis of community stroke educational programs found only two randomized studies in the literature (49). Strict randomization and controlling for confounding variables may be near impossible to implement in cross-country studies of this size. However, future studies should identify confounders of specific importance to FAST Heroes and subsequently design randomized studies that control for selected important variables. Moreover, our study did not assess which program elements were primarily responsible for enhancing parental knowledge, these could incorporate stand-alone assessments of the cartoons, the FAST song, printed material, or informal conversation regarding the program.

Other factors (e.g., television programs, a particular news story, etc.) may have increased parents' stroke-related knowledge independent of the program. We cannot control parents' access to knowledge but consider this a welcomed facilitating ripple effect. Additionally, the SPQ is a variation of the Stroke Action Test (STAT) (43) and is not standardized. However, previous studies have used similar methodologies with success (49). In regard to different number of participants between the parental groups, Group 2 contained data for those who had completed all three assessments (4,202 parents in Group 1 vs. 86 parents in Group 2). We are aware that this smaller number could have caused selection bias. However, the formal launch of the Grand Mission was March 2021, with random starting dates for schools both within and between countries. Therefore, many parents had not reached the 6-month post-implementation mark when we conducted this study. Nevertheless, findings still indicate an overall trend toward sustained knowledge of stroke symptoms. Additionally, the survey was entirely anonymized, thus demographic data for the parent population are non-existent.

Future studies should measure the campaign's impact on the percentage of stroke patients who arrive at the hospital within the time frame to receive medical emergency treatment and changes in the delay of hospital arrival after implementing the FAST Heroes program. Furthermore, focusing on relatives' first reactions and mechanisms behind improving stroke-related knowledge could be obtained by incorporating qualitative questions into evaluations.

Our results demonstrate that the implementation of “Grand Mission” FAST Heroes educational program for 5- to 9-year-old children, which is nested in an ongoing global campaign, has already yielded positive results in the knowledge of thousands of families in different settings and countries. Findings confirm that knowledge about stroke transfers well from children to their families through the FAST Heroes program despite regional differences. A novel result of this study is the affirmed trend toward knowledge retention of parents 6 months after completion of the program. Importantly, parents and teachers consider the program feasible and worthwhile in a wide variety of settings. The results will inform the further rollout of the campaign and future studies. Findings are also likely to be useful in our long-term objective: to get FAST Heroes included in school curriculums to ensure continuous tuition of essential stroke symptoms to families.

## Data Availability Statement

The raw data supporting the conclusions of this article will be made available by the authors, without undue reservation.

## Ethics Statement

The studies involving human participants were reviewed and approved by Committee for Research Ethics of the University of Macedonia (Thessaloniki, Greece) (14/15.06.2020). Written informed consent to participate in this study was provided by the participants' legal guardian/next of kin.

## Author Contributions

All authors listed have made a substantial, direct, and intellectual contribution to the work and approved it for publication.

## Funding

This study received funding from Boehringer Ingelheim (Grant Number 395479, 2019). The funder was not involved in the study design, collection, analysis, interpretation of data, the writing of this article, or the decision to submit it for publication.

## Conflict of Interest

KT, JM, CW, and HP are employed by Boehringer Ingelheim. The remaining authors declare that the research was conducted in the absence of any commercial or financial relationships that could be construed as a potential conflict of interest.

## Publisher's Note

All claims expressed in this article are solely those of the authors and do not necessarily represent those of their affiliated organizations, or those of the publisher, the editors and the reviewers. Any product that may be evaluated in this article, or claim that may be made by its manufacturer, is not guaranteed or endorsed by the publisher.

## Footnotes

^1^ www.fastheroes.com

^2^ https://3gem.com/
