# Supplementary material for: FAST Heroes: Results of Cross-Country Implementation of a Global School-Based Stroke Education Campaign
Source: Front Public Health. 2022 Apr 18;10:849023. doi: 10.3389/fpubh.2022.849023 (PMC9058110; doi:10.3389/fpubh.2022.849023)
Supplement: Supplementary file 1 [file Data_Sheet_1.pdf]

Login Sign Up

HERO TEST

FAST HEROES SAVE LIVES BY IMPROVING KNOWLEDGE.

In our quest to find the best ways to do so we need to measure your baseline knowledge. We will repeat this questionnaire towards the end of the campaign to see if we succeeded in our mission.

Please read through the symptoms listed on the left and tell us what you would do in each case if someone with you is showing that particular symptom.

(Check all options that apply)

Questions	Call a friend or family member for advice	Take a nap and wait for the symptoms to disappear	Consult a family doctor	Call an ambulance	Drive to the closest hospital
01 Persistent headache that builds over time and does not seem to go away.	☐	☐	☐	☐	☐
02 They suddenly could not speak clearly. Their words sound slurred.	☐	☐	☐	☐	☐
03 They may suddenly break into a sweat with cold, clammy skin.	☐	☐	☐	☐	☐
04 They suddenly cannot pick up their glass, as their right arm seems to be sleeping.	☐	☐	☐	☐	☐
05 They are trying to drink some water, but some of the water keeps falling out of one side of their mouth.	☐	☐	☐	☐	☐
06 Discomfort or pain that feels like a tight ache, pressure or squeezing in their chest.	☐	☐	☐	☐	☐

Which of the following do you think could be a symptom of a stroke?

(Check all options that apply)

- [ ] Chest pain
- [ ] Drooping of the face
- [ ] Slurred speech
- [ ] Shortness of breath
- [ ] Arm weakness
- [ ] Goosebumps

Do you know the number to use to call and ambulance?

(Enter the number)

NUMBER

Continue ▶

Terms & conditions   Privacy policy   This campaign is made possible with support by

Department of Education and Social Policy, University of Macedonia, 156 Egnatia Str., P.C.54636, Thessaloniki, Greece, Tel: +30 2310 891630, E-mail: For queries please write to hproios@uom.gr

This campaign was developed by the Department of Education and Social Policy of the University of Macedonia and has been made possible by the support from the Angels Initiative. The Angels Initiative is a Healthcare improvement project aimed at improving stroke care across the world. This is achieved by helping set up new Stroke Ready Hospitals and improving the quality of care in existing Stroke hospitals. The eventual goal is that all stroke patients have access to the same level of care irrespective of where they are in the world. The Angels Initiative is an initiative by Boehringer Ingelheim and is endorsed by the World Stroke Organisation (WSO), The European Stroke Organisation (ESO) and has as partners supporting the initiative the Stroke Alliance For Europe (SAFE) and Medtronic. For more information visit the website at www.angels-initiative.com

Powered by OneLink translations.com
