# Supplementary material for: FAST Heroes: Results of Cross-Country Implementation of a Global School-Based Stroke Education Campaign
Source: Front Public Health. 2022 Apr 18;10:849023. doi: 10.3389/fpubh.2022.849023 (PMC9058110; doi:10.3389/fpubh.2022.849023)
Supplement: Supplementary file 2 [file Data_Sheet_2.pdf]

FAST HEROES - POST PROGRAMME QUESTIONNAIRE

SCHOOL STAFF

Splitter questions:

- Gender:
  - Male
  - Female
  - Other

- Age:
  - Under 30 years
  - 31-40 years
  - 41-50 years
  - 51-60 years
  - 60 years+

- Your education level:
  - Primary School
  - High School
  - University Graduate
  - MSc or PhD

- Location of your school:
  - Town or City with less than 20 thousand inhabitants
  - Town or City with 20 to 100 thousand inhabitants
  - Town or City with 100 to 500 thousand inhabitants
  - Town or City with more than 500 thousand inhabitants

- Have you or do you know a close relative or family friend who has had a stroke?
  - Yes
  - No

  If yes, please give details: ____

Core questions:

1. How many children did you take through the FAST Heroes learning programme?
   a. 1 to 10
   b. 11 to 20
   c. 21 to 30
   d. 30+

2. How did you implement the programme to these children?
   a. In the classroom
   b. Delivered virtually to children working at home
   c. A mixture of a and b

3. How did you hear about the FAST Heroes campaign? (please select your top three)
   a. Direct contact from a campaign partner
   b. Social media

c. Via the World Record attempt
d. Recommended by the Ministry for Educational
e. Recommendation from fellow school/teacher
f. Media coverage
g. Highlighted by a parent at your school
h. Saw it on TV
i. Other (please state)
j. None of the above

4. Why did you decide to help implement the campaign? (please select your top three)
    a. To support an important cause that can make a positive difference
    b. Because it seemed easy to implement
    c. As it can help spread an important message
    d. Because it seemed fun
    e. Because you've been personally affected the impact of stroke
    f. As it looked interesting and rewarding for children
    g. To be part of a global community/campaign
    h. Other (please state)
    i. None of the above

5. Please select an answer for each of the statements below:

    - The campaign sign-up process was relatively easy
        - Strongly Disagree
        - Disagree
        - Neither agree or disagree
        - Agree
        - Strongly agree

    - The support provided ahead of campaign implementation was good
        - Strongly Disagree
        - Disagree
        - Neither agree or disagree
        - Agree
        - Strongly agree

    - The campaign materials were a good quality
        - Strongly Disagree
        - Disagree
        - Neither agree or disagree
        - Agree
        - Strongly agree

    - The children enjoyed taking part in the campaign
        - Strongly Disagree
        - Disagree
        - Neither agree or disagree
        - Agree
        - Strongly agree

- Children passed on the messages to their family members
  - Strongly Disagree
  - Disagree
  - Neither agree or disagree
  - Agree
  - Strongly agree

- The campaign can help save lives
  - Strongly Disagree
  - Disagree
  - Neither agree or disagree
  - Agree
  - Strongly agree

- I would recommend the campaign to others
  - Strongly Disagree
  - Disagree
  - Neither agree or disagree
  - Agree
  - Strongly agree

6. How much time did you spend in your class implementing the programme?
   a. Less than 5 hours
   b. 5 hours
   c. More than 5 hours

7. In which ways did the children learn through the programme? (please select your top three)
   a. They learnt about ways for helping loved ones
   b. They learnt to recognise the signs of a stroke
   c. They learnt, or were reminded about, the emergency services number
   d. They learnt how to react in an emergency situation
   e. Other (please state)
   f. None of the above

8. What do you think are the best elements of the campaign materials? (please select your top three)
   a. The characters/FAST Heroes
   b. The animated films
   c. The educational workbooks
   d. The online e-books
   e. The way they make learning fun
   f. The fact it is all free
   g. The online resources e.g. video-computer games
   h. None of the above

9. What are the main symptoms of a stroke? (please tick all that apply)
   a. Chest pain
   b. Drooped face/mouth
   c. Swollen limbs
   d. Weak arm
   e. Breathing problems

    f. Slurred speech
    g. None of the above

10. If you see someone having a stroke, what should you do straight away? (Select one answer)
    a. Call a doctor
    b. Get him / her to the nearest hospital
    c. Call an ambulance
    d. Tell him / her to get rest
    e. Call a relative or friend to ask for advice
    f. Wait and see how they feel in an hour
    g. Nothing

11. To what extent do you agree with the following statements?

    - 'Getting urgent medical treatment for a stroke can have a big impact on somebody's recovery'
        i. Strongly Disagree
        ii. Disagree
        iii. Neither agree or disagree
        iv. Agree
        v. Strongly agree

    - 'The FAST Heroes campaign has increased my confidence about what to do if somebody has a stroke'
        - Strongly Disagree
        - Disagree
        - Neither agree or disagree
        - Agree
        - Strongly agree

12. What is the most appropriate number used to call an ambulance in case of a stroke in your country?
    a. 125
    b. 112
    c. 111
    d. 101

13. Did you receive any positive feedback from parents about their child's participation in the campaign?
    a. Yes. If so, can you please specify?
    b. No. If no, is there anything you think we can change to improve the campaign?
    c. Not sure

14. Would you be happy to be contacted to provide additional comment or to feature in future campaign materials?
    - Yes (field to provide name and contact details)
    - No

15. Please feel free to add anything that you would like to share with us (optional):

PARENTS

Splitter questions:

- Gender:
  - Male
  - Female
  - Other

- Age:
  - Under 30 years
  - 31-40 years
  - 41-50 years
  - 51-60 years
  - 60 years+

- How many children do you have?
  - One
  - Two
  - Three or more

- What age is your child who took part in the FAST Heroes campaign? If you have more than 1 child, please choose the age range for all that took part
  - 4
  - 5
  - 6
  - 7
  - 8
  - 9+

- Do your parents or in-laws live with you?
  - Yes
  - No

- Your education level:
  - Primary School
  - High School
  - University Graduate
  - MSc or PhD

- Location of your school:
  - Town or City with less than 20 thousand inhabitants
  - Town or City with 20 to 100 thousand inhabitants
  - Town or City with 100 to 500 thousand inhabitants
  - Town or City with more than 500 thousand inhabitants

- Have you or do you know a close relative or family friend who has had a stroke?
  - Yes
  - No

  If yes, please give details: ____

Core questions:

1. How did you know your child was taking part in the FAST Heroes campaign? (please select your top three)
    a. My child told me
    b. My child's teacher told me
    c. The school sent me an email (or other contact) to inform me
    d. Another parent at the school told me
    e. Other (please state)
    f. None of the above

2. Which of the following do you agree with regarding the campaign? (please select your top three)
    a. It's an important cause that can make a positive difference
    b. It's easy to take part
    c. It can help spread an important message
    d. It's a fun and rewarding campaign for children
    e. Other (please state)
    f. None of the above

3. Please select an answer for each of the statements below:

    - The campaign materials were a good quality
        - Strongly Disagree
        - Disagree
        - Neither agree or disagree
        - Agree
        - Strongly agree

    - My child/children enjoyed taking part in the campaign
        - Strongly Disagree
        - Disagree
        - Neither agree or disagree
        - Agree
        - Strongly agree

    - My child/children seemed to understand the messages being communicated through the campaign
        - Strongly Disagree
        - Disagree
        - Neither agree or disagree
        - Agree
        - Strongly agree

    - My child/children passed on the messages to our family members
        - Strongly Disagree
        - Disagree
        - Neither agree or disagree
        - Agree
        - Strongly agree

- The campaign can help save lives
  - Strongly Disagree
  - Disagree
  - Neither agree or disagree
  - Agree
  - Strongly agree

- I would recommend the campaign to others
  - Strongly Disagree
  - Disagree
  - Neither agree or disagree
  - Agree
  - Strongly agree

4. In which ways do you think your child learnt through the programme? (please select your top three)
   a. They learnt about ways for helping loved ones
   b. They learnt to recognise the signs of a stroke
   c. They learnt, or were reminded about, the emergency services number
   d. They learnt how to react in an emergency situation
   e. Other (please state)
   f. None of the above

5. Did you personally learn more about stroke and the importance of calling an ambulance straight away as a result of the campaign?
   a. Strongly Disagree
   b. Disagree
   c. Neither agree or disagree
   d. Agree
   e. Strongly agree

6. Which of the following did your child do as part of their involvement in the campaign? (please select your top three)
   a. They showed me printed campaign materials they'd been given
   b. They showed me the FAST Heroes website
   c. They nominated two Grandparents or family members to involve in the campaign
   d. They passed on the message/materials to their Grandparents/family members
   e. They took part in the 'Superhero Selfie' World Record attempt
   f. None of the above

7. What do you think are the best elements of the campaign materials? (please select your top three)
   a. The characters/FAST Heroes
   b. The animated films
   c. The educational workbooks
   d. The online e-books
   e. The way they make learning fun
   f. The fact it is all free
   g. The online resources e.g. video-computer games
   h. None of the above

8. What are the main symptoms of a stroke? (please tick all that apply)
    a. Chest pain
    b. Drooped face/mouth
    c. Swollen limbs
    d. Weak arm
    e. Breathing problems
    f. Slurred speech
    g. None of the above

9. If you see someone having a stroke, what should you do straight away? (Select one answer)
    a. Call a doctor
    b. Get him / her to the nearest hospital
    c. Call an ambulance
    d. Tell him / her to get rest
    e. Call a relative or friend to ask for advice
    f. Wait and see how they feel in an hour
    g. Nothing

10. To what extent do you agree with the following statements?

    - 'Getting urgent medical treatment for a stroke can have a big impact on somebody's recovery'
        i. Strongly Disagree
        ii. Disagree
        iii. Neither agree or disagree
        iv. Agree
        v. Strongly agree

    - 'The FAST Heroes campaign has increased my confidence about what to do if somebody has a stroke'
        - Strongly Disagree
        - Disagree
        - Neither agree or disagree
        - Agree
        - Strongly agree

11. What is the most appropriate number used to call an ambulance in case of a stroke in your country?
    a. 125
    b. 112
    c. 111
    d. 101

12. Is there anything you think we can change to improve the campaign in the future? (open text)

13. Would you be happy to be contacted to provide additional comment or to feature in future campaign materials?
    - Yes (field to provide name and contact details)
    - No

14. Please feel free to add anything that you would like to share with us (optional):
